# Sintering Temperature Effect on Structural and Optical Properties of Heat Treated Coconut Husk Ash Derived SiO_2_ Mixed with ZnO Nanoparticles

**DOI:** 10.3390/ma13112555

**Published:** 2020-06-04

**Authors:** Muhammad Fahmi Anuar, Yap Wing Fen, Mohd Hafiz Mohd Zaid, Nur Alia Sheh Omar, Rahayu Emilia Mohamed Khaidir

**Affiliations:** 1 Department of Physics, Faculty of Science, University Putra Malaysia, UPM Serdang, Selangor 43400, Malaysia; mhmzaid@upm.edu.my; 2 Functional Devices Laboratory, Institute of Advanced Technology, University Putra Malaysia, UPM Serdang, Selangor 43400, Malaysia; nuralia.upm@gmail.com (N.A.S.O.); rahayuemilia.upm@gmail.com (R.E.M.K.)

**Keywords:** coconut husk ash, ZnO, SiO_2_, crystal, optical, structural

## Abstract

In this work, waste coconut husk ash was used to prepare a ZnO-SiO_2_ composite. Solid-state technique was used to fabricate the composite due to its producibility, simple procedure as well as lower production cost. At high sintering temperatures ranging from 600 °C to 1000 °C, the X-ray diffraction (XRD) peaks of the Zn_2_SiO_4_ showed high intensity, which indicated high crystallinity. Furthermore, the formation of broad bands of ZnO_4_, Si-O-Si, and SiO_4_ were detected by Fourier transform infrared (FTIR) spectroscopy and the bands became narrower with the increment of sintering temperature. Besides, the morphological image from field emission scanning electron microscopy (FESEM) showed the formation of densely packed grains and smooth surface composite with the increase of sintering temperature. Upon obtaining the absorbance spectrum from Ultraviolet–Visible (UV–Vis) spectroscopy, the optical band gap was calculated to be 4.05 eV at 1000 °C. The correlation between the structural and optical properties of ZnO-SiO_2_ composite was discussed in detail.

## 1. Introduction

In recent years, the demands of ‘green’ environmentally friendly processes are growing higher and are much needed as a result of environmental concerns [1,2]. The increasing demand for using the biosynthesis method is due to its advantages as it is simple, environmentally friendly, cost-effective, easy to reproduce, and often more stable [3]. A number of agricultural wastes have been studied that are able to produce biosynthesis materials such as SiO_2_ [4,5,6,7,8]. Among the prominence agricultural sources of silica, coconut husk is believed to have great potential in the optical industry due to the fact that 91.76% of silica can be extracted from coconut husk ash (CHA) [9]. In addition, different types of silica can be extracted by using chemical treatments that are flexible and can be used in different kinds of applications [10]. Despite the advantages, there have been fewer studies done on CHA as more focus has been given to its ability as a good absorbent material toward wastewater treatment problems. Undoubtedly, these biosynthesis materials have great potential to be utilized, for example, as a ZnO dopant to modify its properties.

Zinc oxide (ZnO), which in white powder is an inorganic compound that is insoluble in water, is commonly used as an additive in a number of materials and products such as plastics, rubbers, and ceramics. It is also known for its wide-band gap and is used widely in various applications including devices in ultraviolet light-emitting diodes (UV LEDs), blue laminating devices, and UV lasers [11,12]. ZnO based material has also caught the attention of researchers because of its non-toxicity, environment-friendly, and low-cost, causing it to be highly valued by industry and researchers alike [13]. Furthermore, it is also known that doping with different types of materials can alter and modify its promising properties [14,15,16]. However, ZnO is unstable in most specialized applications and causes red-shift in absorption spectra [17]. Obviously, new synthesis methods that utilize ‘green’ methodology are preferable as some researchers have proven them to be safer and they can be extracted from plant-based materials.

In the past decades, numerous studies have been conducted on a mixture of ZnO and SiO_2_. SiO_2_ has generally been used as a host material for ZnO nanoparticles due to its chemical and physical stability properties [18]. Traditionally, zinc silicate forms when heat treatment is applied at a certain temperature [19,20]. Zinc silicate is one of the most promising phosphors materials known today because of its excellent luminescence properties in blue, green, and red spectral regions [21]. Wide energy band gap (5.5 eV), exceptional and high luminescent efficiency are also the benefitting factors that are vital in optoelectronic devices. In addition, zinc silicate has been established as a good host material and is suitable for applications in cathode ray tubes, plasma display panels, laser crystal, and electroluminescent devices [22,23,24]. Furthermore, the optical properties of zinc silicate can also be enhanced and modified by doping with different types of metals and applying heat treatment for preferred properties in different applications.

Moreover, the physical, chemical, and optical properties of ZnO-SiO_2_ are dependent on the preparation process and starting materials [20]. Consequently, various methods to synthesize ZnO-SiO_2_ have been developed such as hydrothermal [25], sol-gel [23,26], spray pyrolysis [17], and the conventional solid-state method [27,28]. The solid-state method is much simpler, and the materials can be produced in large quantities compared to other methods that require complicated steps, long preparation periods, and high equipment costs.

To the best of our knowledge, there have been limited studies on producing ZnO-based composites based on agricultural waste, especially coconut husk using the solid-state method. Hence, the aim of this work was to prepare a ZnO-SiO_2_ composite by incorporating silica derived from coconut husk into ZnO, and to investigate their structural and optical properties subjected to sintering temperature.

## 2. Materials and Methods

The sample of coconut husk ash (CHA) used in the study was obtained from the process of burning the coconut husk at 700 °C and treating as described in the previous report [10]. CHA was used as the source of SiO_2_ with 91.76% purity after treatment, which was confirmed by XRF as reported. In this study, ZnO nanoparticles (US Research Nanomaterials Inc., Houston, TX USA, 99%, 10–30 nm) were added with CHA to form a mixture of 20.0 g with the ratio of 1:1 using a ball milling jar for 24 h. After that, the mixed composition was pressed at 5 tons pressure to form a pellet. Next, the pellet was sintered in an alumina crucible at 600 °C to 1000 °C with a constant heat rate for 2 h.

X-ray diffraction (XRD) analysis was carried out by using a Philips X’Pert HighPro PANanalytical Diffractometer (Malvern Pananalytical, Almelo (The Netherland) and Malvern (UK)) with a copper (Cu) anode and Cu k-α radiation with wavelength 1.5406 Å operated at 40 mA and 40 kV. A Thermo Nicolet Nexus FTIR (Thermofisher Scientific, Waltham, MA, US) was used in the range of 280 to 4000 cm^−1^ to determine the bonds in the composite. Nova NanoSEM 30 (FEI, Hillsboro, OR, USA) was used to study the morphological structure of the composite sample with a 50,000 magnification level. Furthermore, the optical properties were studied using a UV-3600 Shimadzu (Shimadzu, Kyoto, Japan).

## 3. Results

**Figure 1:**
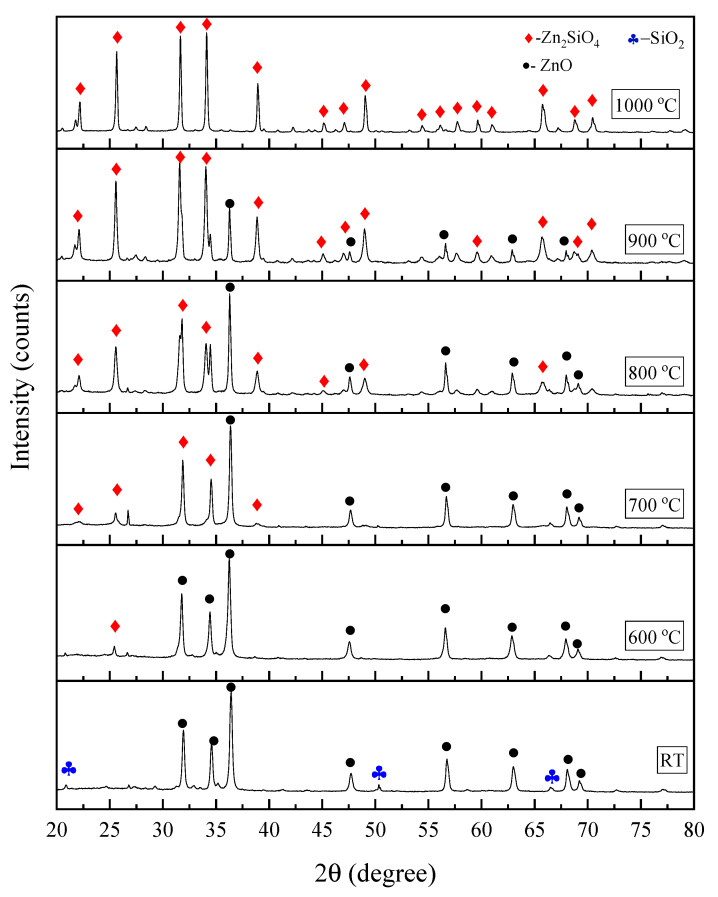
X-ray diffraction (XRD) spectrum of the ZnO-SiO_2_ composite sintered at different temperatures.

The phase identification of the sample was carried out by using XRD analysis and X’Pert Highscore Plus software was used to identify the diffraction peaks obtained in Figure 1. ZnO (JCPDS 14-0653) and SiO_2_ (JCPDS 82-0511) were identified on the peaks of the unsintered (RT) composite. At 600 °C, the zinc silicate (Zn_2_SiO_4_) diffraction peak appeared (JCPDS 37-1485) at 2θ = 25.44°. The zinc silicate had a rhombohedral structure with lattice parameter a = b = 13.938 (Å) and c = 9.3100 (Å), space group of R$\bar{3}$, and space group number of 148, as also reported in previous research [6,29,30]. Some zinc oxide (ZnO) (JCPDS 14-0653) peaks also appeared at 2θ = 31.78°, 34.44°, 36.26°, 47.58°, 56.59°, 62.95°, 68.08°, and 69.06°. As the temperature increased to 700 °C and 800 °C, more Zn_2_SiO_4_ peaks appeared and less ZnO peaks existed in the spectrum. Based on the XRD spectrum, the zinc silicate diffraction peaks appeared at 2θ = 22.02°, 25.56°, 31.88°, and 38.96° after a sintering temperature of 700 °C, and at 800 °C, it appeared at 2θ = 45.09°, 48.99°, 65.07°. Next, the diffraction peaks of ZnO located at 2θ = 36.26°, 47.58°, 56.69°, 62.95°, and 68.08° started to disappear at the higher temperature of 900 °C and was completely gone at 1000 °C. This phenomenon indicated greater fusion of ZnO and SiO_2_ to form zinc silicate at 900 °C and was completed at 1000 °C. Upon reaching the sintering temperature of 1000 °C, the zinc silicate diffraction peaks located at 2θ = 22.19°, 25.66°, 31.65°, 34.12°, 38.94°, 45.14°, 47.07°, 49.03°, 54.39°, 56.14°, 57.70°, 59.63°, 60.97°, 65.73°, 68.77°, and 70.45° became more intensified and sharper due to the higher sintering temperature increasing the crystallinity of the zinc silicate [31,32].

**Figure 2:**
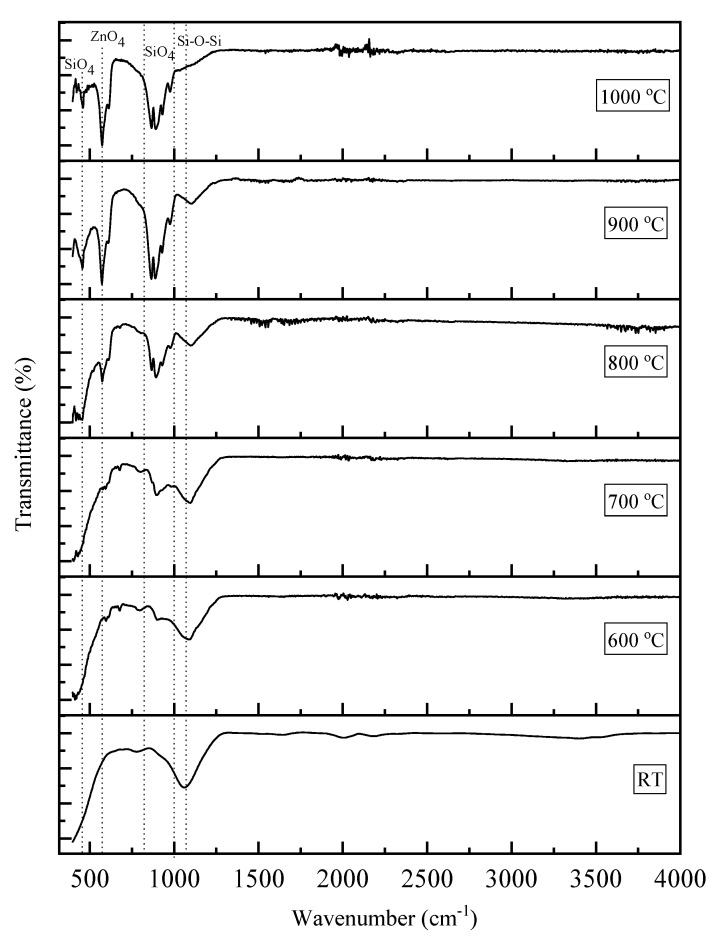
Fourier transform infrared (FTIR) spectra of ZnO-SiO_2_ at different sintering temperatures.

The FTIR spectrum of the ZnO-SiO_2_ composite derived from coconut husk ash sintered at several temperatures was observed in the range of 400–4000 cm^−1^, as illustrated in Figure 2. The pre-sintering sample (RT) showed a broad absorption band of Si-O-Si asymmetric stretching vibration, which resides at 1060 cm^−1^, probably due to the change in bonding around the tetrahedral SiO_4_. These results are in a good agreement with previous studies [29,33]. Upon the sintering temperatures of 600 °C and 700 °C, a few weak absorption bands were observed at wavenumbers 570 cm^−1^ and 900 cm^−1^. These two bands can be attributed to the ZnO_4_ symmetric stretching and SiO_4_ asymmetric stretching, respectively [29,34,35,36]. At a temperature of 800 °C, a band attributed to ZnO_4_, was observed at 450 cm^−1^ [36]. Furthermore, from 900 °C up to 1000 °C, the broad peaks of ZnO_4_ (570 cm^−1^) and SiO_4_ (900 cm^−1^) became narrower and stronger as the sintering temperature increased, since high temperature increased the crystallinity of the composite, as indicated by the XRD analysis. In a nutshell, the observed appearances of the band attributed to SiO_4_ and ZnO_4_ indicate the successful formation of the crystalline ZnO-SiO_2_ composite, especially starting at a temperature of 800 °C and above, which was also supported by the XRD spectrum.

**Figure 3:**
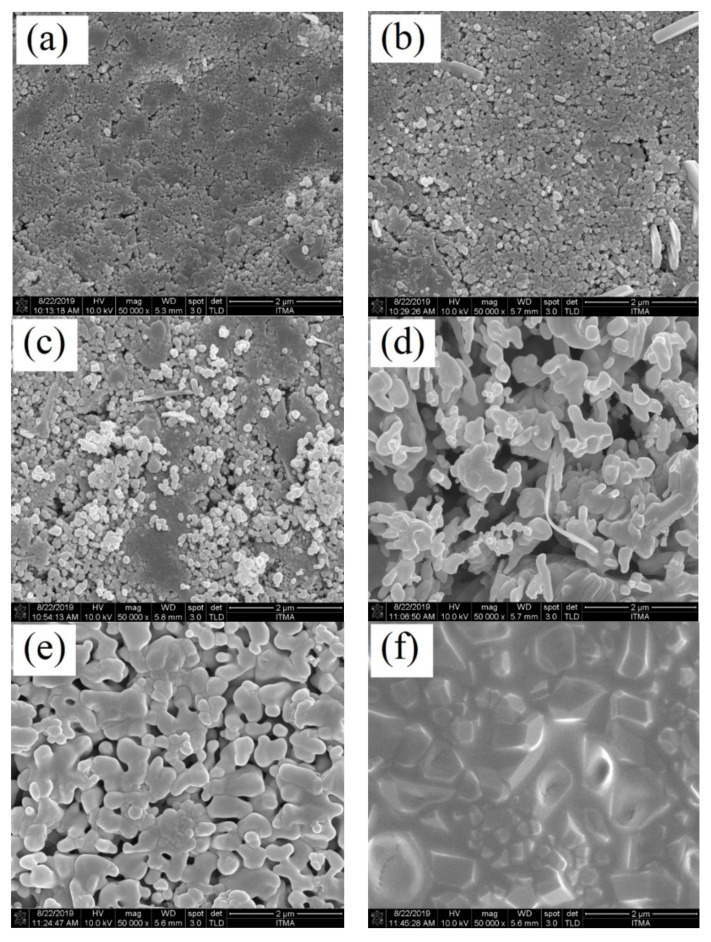
Field emission scanning electron microscopy (FESEM) images of composite sample ZnO-SiO_2_ at (**a**) RT, (**b**) 600 °C, (**c**) 700 °C, (**d**) 800 °C, (**e**) 900 °C, and (**f**) 1000 °C.

The microstructure images of the ZnO-SiO_2_ composite that was heat treated at various sintering temperatures of 600–1000 °C were studied using field emission scanning electron microscopy (FESEM) at a 50,000 magnification level. As shown in Figure 3, it was observed that the composite sample did not have a uniform shape or structure. Additionally, it can be observed from the images that the particle size distribution of the composite samples was irregular and not consistent when it was not heat-treated. There was also a rod-like formation observed on the untreated (RT) composite at 600 °C that originated from the coconut husk ash structure [10]. Based on Figure 3, the average grain sizes were measured and tabulated in Table 1. The average grain sizes of the sample at RT was measured to be 72.23 nm and increased to 94.23 nm after heat-treated at 600 °C. Next, at a temperature of 700 °C, the average grain sizes increased to 121.37 nm and aggregated due to an increase in the sintering temperature. Furthermore, the samples sintered at 800 °C and 900 °C showed average grain sizes of 299.37 nm and 326.83 nm, respectively, due to the increase in grain growth and crystallinity of the sample that was caused by a higher sintering temperature. However, the sample sintered at 900 °C showed densely packed grains of different sizes with less porosity present in the sample. Particles in the samples started to form necks between each other and agglomerated to form bigger particles. The sample sintered at 1000 °C showed a smooth surface with well-distinct boundaries in the images and the average grain sizes were measured at 515.70 nm. The image indicated that the composite sample was well-crystallized after being sintered at 1000 °C. The formation of rhombohedral-like particles also confirmed the formation of the high crystallinity of Zn_2_SiO_4_ when sintered at high temperature.

It can also be observed from the images, where the particle grain sizes increased with the sintering temperatures as a result of grain growth. Grain growth is known as a temperature-dependent phenomenon and thus the composite samples increased in sizes along with the sintering temperature [37]. The grain growth with well-distinct characteristics with the sintering temperatures suggested that sintering also increased the crystallization of the composite sample of ZnO-SiO_2_ and produced high crystallinity at 1000 °C.

**Figure 4:**
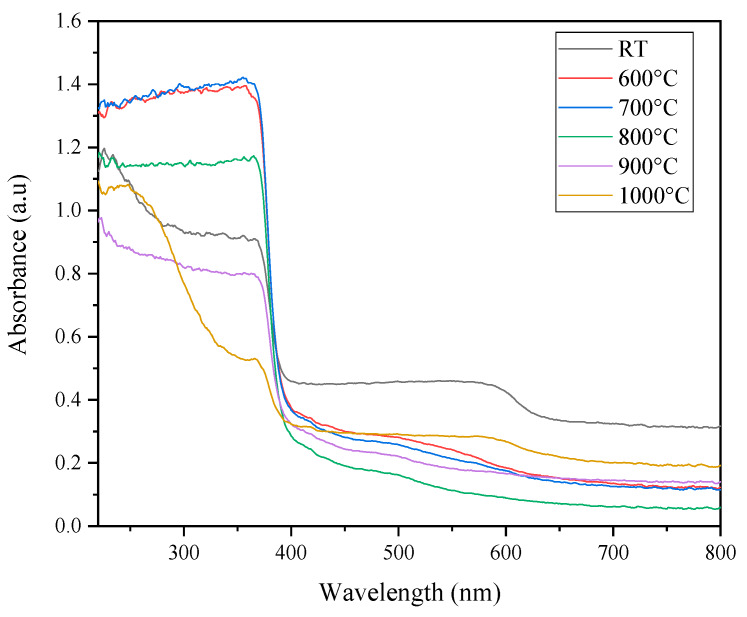
Optical absorbance spectra for the ZnO-SiO_2_ composite sintered at different sintering temperatures.

For the optical properties, the absorption spectra of ZnO-SiO_2_ sintered at various temperatures are shown in Figure 4. From the absorbance spectrum, a sharp absorption edge was observed at a wavelength of about 400 nm, indicating that the absorption properties of the ZnO-SiO_2_ composite at room temperature occurred in the UV region. When the prepared composite was sintered at 600 °C to 1000 °C, the absorbance intensity starting to decrease with the increase in temperature. This occurrence was due to the deformation of the ZnO hexagonal structure in the sample and started to form a zinc silicate composite structure. Furthermore, the absorption edge of the sintered samples shifted to a longer wavelength (red-shifted) when at 600 °C to 800 °C. This occurrence suggests that the crystallization process between temperatures of 600 °C and 800 °C enhanced the absorption edge toward the red-shift. However, when the sintering temperatures were 900 °C and 1000 °C, the absorption edge shifted back to the lower wavelength, which can be attributed to the high crystallinity of the zinc silicate composite sample [36].

The optical band gap of all the ZnO-SiO_2_ composite samples was determined by using the following relation between absorption coefficient, α, and photon energy, *hν* [38]:

(1) $$\alpha =\frac{k{\left[hv-E_{\mathrm{gap}}\right]}^{n}}{hv}$$

By rearranging Equation (1) above, it gives:

(2) $${\left(\alpha hv\right)}^{1/n}=hv-E_{\mathrm{gap}}$$

where *ν* is the frequency; *h* is the Plank constant; *n* is the type of transition; and *E*_gap_ is the optical band gap. In this work, *n* = 1/2 was used as the direct allowed transition, and by inserting the value of *n* into Equation (2), it becomes:

(3) $${\left(\alpha hv\right)}^{2}=hv-E_{\mathrm{gap}}$$

**Figure 5:**
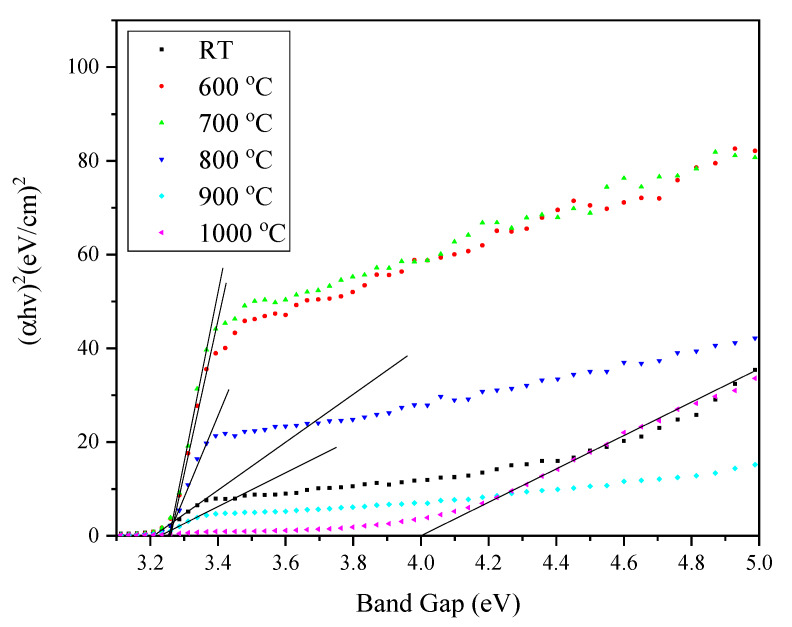
The optical band gap of the ZnO-SiO_2_ composite at different sintering temperatures.

By using the Equation (3) relation, the optical band gap can be obtained by extrapolating the linear fitted region of the graph of (*αhν*)^2^ against *hv* to the position at which (*αhν*)^2^ = 0. The variations of (*αhν*)^2^ of ZnO-SiO_2_ as a function of photon energy sintered at different temperatures are shown in Figure 5.

When the sintering temperature increased, the amount of vibration energy provided to the atom increased and caused the interatomic spacing between the atoms to weaken. Consequently, a weak interatomic spacing allowed weak energy to break the bond and caused the gap between the conduction and valence band to shrink [39]. Inversely, the band gap value promptly increased when the temperature reached 1000 °C. The high crystallinity of the composite at 1000 °C caused the band gap value to increase rapidly to 4.05 eV [40]. The improvement of the composite crystallinity allowed the reduction of delocalized states, resulting in a composite with less defects, and thus increased the optical band gap value [39,41]. The formation of the rhombohedral phase (Zn_2_SiO_4_) was also the justification of the phenomenon that occurred at a sintering temperature of 1000 °C. Consequently, the absorption edge became broad and increased the optical band gap values of the ZnO-SiO_2_ composite due to the comparatively large band gap of zinc silicate.

## 4. Conclusions

The composite ZnO-SiO_2_ was prepared and the zinc silicate started to form at a temperature of 600 °C and increased in the diffraction peak intensity at a higher temperature until 1000 °C. The formation of bands assigned to ZnO_4_ and SiO_4_ was detected in the FTIR spectrum and became stronger at 1000 °C. Next, the FESEM images of the ZnO-SiO_2_ composite revealed that at 1000 °C, a well-distinct crystal formation with densely packed grains was observed, indicating the high crystallinity of ZnO-SiO_2_. Moreover, the optical band gap of the ZnO-SiO_2_ composite at room temperature was 3.22 eV, and this value increased to 4.05 eV at 1000 °C due to the formation of highly crystalline zinc silicate composite. Finally, with all the evidence obtained, we can conclude that zinc silicate was formed after the heat treatment. Zinc silicate is known to be a good host material for doping with rare-earth ions and transition metals. Therefore, it is of interest to further expand this study by exploring the possibility of turning this zinc silicate from coconut husk into a phosphor material.

## Figures and Tables

**Table 1:** Average grain sizes of ZnO-SiO_2_ at different sintering temperatures.

Sintering Temperature (°C)	Average Grain Sizes (nm)
RT	72.23
600	94.23
700	121.37
800	299.37
900	326.83
1000	515.70
